# Prejudice against Doctors

**Published:** 1914-11

**Authors:** M. M. Carrick


### Prejudice Against Doctors.

BY M. M. CARRICK, M. D.

Despite universal complaints of the layman about the fallibility of the members of the medical profession, with the possible exception of his own family physician, there are a few outstanding facts to be considered in favor of that much maligned individual—the doctor.

Unless we are generous enough to accept the service of the church, which throughout its history has dispensed much practical charity and given much gratuitous service to the cause of humanity, there is no other organization that has ever maintained, or so scrupulously adhered to so high a code of ethics as the medical fraternity. In fact, there is scarcely another profession that requires such careful, exacting, continuous training as that of a physician. When he is graduated from his medical school he is, of course, qualified to practice medicine; but whether he takes up general practice, surgery or becomes a specialist in one of the numerous branches of the profession, the only way that he can possibly succeed is by keeping abreast of the times. This means constant study every spare moment, to say nothing of the post-graduate at home and abroad.

The medical man must come in contact with master minds in his chosen profession and learn all he can about the newest discoveries in the realm of materia medica—the latest methods of anesthesia, the last word in surgery and orthopedic applications, the uses of serums, toxins and every known aid to prevent disease and relieve human suffering. In other words, there is always something new to learn—some amplification or scientific equipment necessary. Discoveries fairly tumble over one another, and the medical man who is conscientiously meeting the exacting requirements of his vocation has but little time to keep pace with them all.

The profession of medicine calls for social service of the highest sort. That this was recognized from earliest time is shown by the Code of Ethics adopted by the American Medical Association in 1847. After referring to physicians as conservators of public health, the code declares:

"It is a delicate and noble task by the judicious application of public hygiene to prevent disease and lengthen life, thus increasing the productive energy without assuming the office of moral and religious training to add to the civilization of an entire people."

In the "Dark Ages" of our calling, but a few years back, physicians treated one another with inconsideration. Rivalry was rampant. In due time, there came a reaction, and the profession began to feel the effects of the enormity of the evil, for which its own members were responsible. Then there came a change in the mental attitude of physicians generally. By degrees the little leaven nearly leavened the whole lump, until the profession has become a harmonious one with the exception of a few cases,—isolated cases, however, who still work harm to the profession to which they have sworn fealty. The standard of medicine is thus lowered in the eyes of the public, though the individual may stand high with his patients.

This is far from what our mentors intended when they gave us the Code of Ethics referred to.

Can a layman think of any other profession that systematically works against its own interests? We who have hitherto lived by the prevalence of disease now find it worth while, for the sake of humanity and social progress, to labor for the conservation of public health, even though we know we are decreasing our incomes by the preventive measures we employ.

We have found it much easier and safer to prevent than to try to cure disease, and, as ours is a humanitarian calling, we must live up to its requirements. The public that profits by our efforts to prevent disease should extend a more generous measure of co-operation, if for no other reason than that all public health measures will be brought to a realization earlier if there is co-operation between the public and the medical profession. That there is a lack of support in this direction at the present time is due to a misconception on the part of the public with reference to the purpose of the physician. When this is generally understood, there should be no further obstruction to the unselfish work the physician is doing.

---

Dr. H. L. McNeil, who has practiced medicine in Houston for the past two years, has gone to Galveston to take up his duties as a member of the faculty of the medical college of the University of Texas. Dr. McNeil's friends are elated over his appointment.

